# An Ab Initio QM/MM Study of the Electrostatic Contribution to Catalysis in the Active Site of Ketosteroid Isomerase

**DOI:** 10.3390/molecules23102410

**Published:** 2018-09-20

**Authors:** Xianwei Wang, Xiao He

**Affiliations:** 1 College of Science, Zhejiang University of Technology, Hangzhou 310023, Zhejiang, China; wxw263@163.com; 2 Shanghai Engineering Research Center of Molecular Therapeutics and New Drug Development, School of Chemistry and Molecular Engineering, East China Normal University, Shanghai 200062, China; 3 NYU-ECNU Center for Computational Chemistry at NYU Shanghai, Shanghai 200062, China

**Keywords:** electric field, hydrogen-bond network, vibrational Stark effect, enzyme catalysis

## Abstract

The electric field in the hydrogen-bond network of the active site of ketosteroid isomerase (KSI) has been experimentally measured using vibrational Stark effect (VSE) spectroscopy, and utilized to study the electrostatic contribution to catalysis. A large gap was found in the electric field between the computational simulation based on the Amber force field and the experimental measurement. In this work, quantum mechanical (QM) calculations of the electric field were performed using an ab initio QM/MM molecular dynamics (MD) simulation and electrostatically embedded generalized molecular fractionation with conjugate caps (EE-GMFCC) method. Our results demonstrate that the QM-derived electric field based on the snapshots from QM/MM MD simulation could give quantitative agreement with the experiment. The accurate calculation of the electric field inside the protein requires both the rigorous sampling of configurations, and a QM description of the electrostatic field. Based on the direct QM calculation of the electric field, we theoretically confirmed that there is a linear correlation relationship between the activation free energy and the electric field in the active site of wild-type KSI and its mutants (namely, D103N, Y16S, and D103L). Our study presents a computational protocol for the accurate simulation of the electric field in the active site of the protein, and provides a theoretical foundation that supports the link between electric fields and enzyme catalysis.

## 1. Introduction

Although it is still a controversial issue on the origin of the tremendous catalytic power of enzymes, it has been commonly accepted that the electrostatic field plays a key role in the enzyme’s high catalytic proficiency [1,2]. The active site of enzymes would provide a quite different preorganized electrostatic environment from solvent that preferentially stabilizes the charge distribution of the transition state, thus reducing the energy barrier of reaction and enhancing the reaction rate. A recent work [3] by Aragonès et al. demonstrated that the applied electric field could directly enhance the Diels–Alder reaction rate. Moreover, the fact that the catalytic rate of enzyme correlates with the magnitude of the electric field at the active site of enzyme has been experimentally verified using vibrational Stark effect (VSE) spectroscopy in the studies on some proteases [4,5,6,7]. Hence, the accurate description of the electric field in proteins is important for analyzing the structural functions of enzymes.

The technique of VSE spectroscopy has been widely utilized to measure the electric field in proteins such as myoglobin [8,9,10,11], human aldose reductase (hALR2) [12,13,14,15,16], ketosteroid isomerase (KSI) [5,6] and some other proteases [4]. C=O and nitrile groups are often used as ideal vibrational probes to deliver unique vibrations to the specific site of interest in proteins. In many cases, the observed frequency shift of the probe group $\Delta {\tilde{v}}_{probe}$ and the change of the electric field $\Delta {\vec{F}}_{protein}$ (resulting from point mutations as well as a change in the solvent environment and/or the conformational state of a protein) have a linear relationship as follows [17,18,19]:

(1) $$hc\Delta {\tilde{v}}_{probe}=-\Delta {\vec{u}}_{probe}\cdot \Delta {\vec{F}}_{protein}$$

where *h* is the Planck constant and *c* is the speed of light. $\left|\Delta {\vec{u}}_{probe}\right|$ is the Stark tuning rate of the probe, which is a constant in many cases, and can be obtained by calibrating in a well-defined electric field [7]. It is worth noting that Equation (1) may not be strictly correct for some nitrile-containing probe molecules in the case that the hydrogen-bonding interaction with the nitrile is involved [18]. However, some previous studies [18,19,20] have shown that the linear relationship between the observed frequency shift of the probe group $\Delta {\tilde{v}}_{probe}$ and the change of electric field $\Delta {\vec{F}}_{protein}$ still holds in both hydrogen-bonding and non-hydrogen-bonding environments for carbonyl vibrational probe.

**Figure 1:**
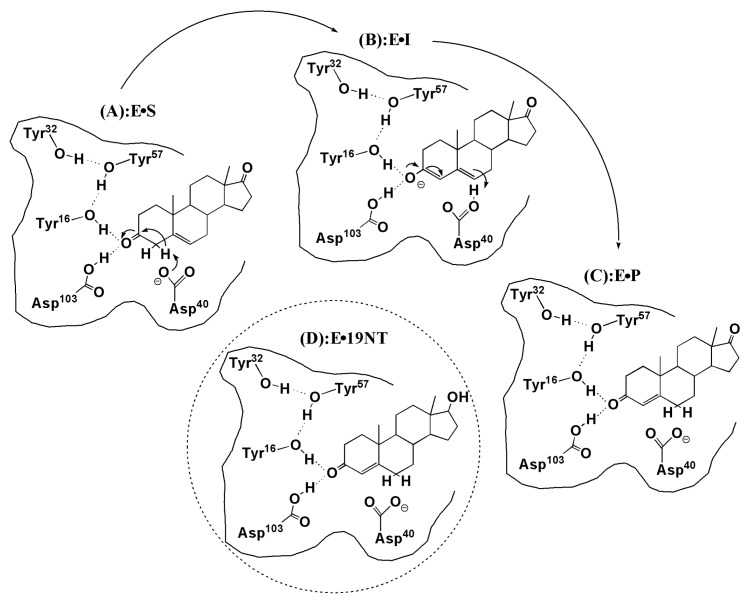
Mechanism of the isomerization reaction catalyzed by ketosteroid isomerase (KSI) and the product-like inhibitor, 19-nortestosterone (19-NT). (**A**) E·S is the enzyme (E) and 5-androstene-3, 17-dione (S, substrate). (**B**) The intermediate state (I), the formation of an enolate with a negative charge, is stabilized by the H-bond network formed by the Tyr16 and Asp103. (**C**) E·P is the enzyme and 4-androstene-3, 17-dione (P, product). (**D**) The complex between the enzyme (KSI) and inhibitor 19-NT (E·19-NT) that has the same bound state as the natural substrate.

**Figure 2:**
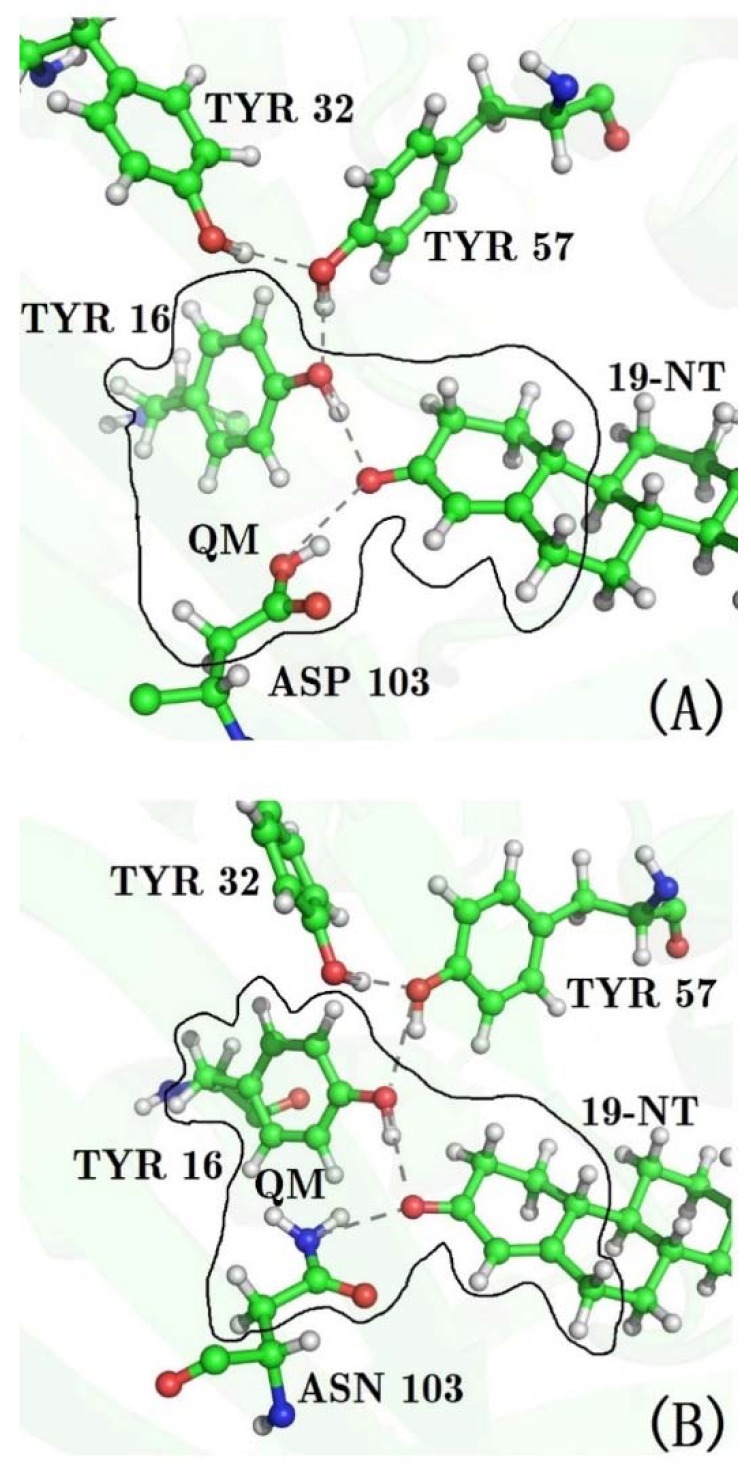
Structures of the hydrogen bonding network of the active site of KSI. (**A**) wild type (**B**) D103N mutant. The side chains of residues 16, 103, and the ring of 19-NT, which the C=O group bonds to, were partitioned into the QM region in the QM/MM MD simulations.

KSI is a small, proficient enzyme with a high catalytic efficacy. Its mechanism and catalytic strategies have been widely studied in biochemistry because of its ultrahigh unimolecular rate constants [21,22]. In steroid biosynthesis and degradation, KSI catalyzes the isomerization of its steroid substrate by altering the position of the C=C double bond (see Figure 1). The Asp40 of KSI will abstract a nearby α proton of the steroid substrate and initiate a rehybridization that converts the adjacent ketone group to a charged enolate intermediate (E·S ⇌ E·I, Figure 1a,b). The intermediate state (IS) is normally unstable and very slow to form because its free energy barrier is high. Considering that the carbonyl bond of the substrate undergoes a charge rearrangement in the formation of IS, a strong electric field with a specific direction is therefore expected to stabilize the charge distribution of the IS. The X-ray structure of KSI [6] shows that an extended hydrogen-bonding network that involves four residues (TYR16, TYR32, TYR57, and ASP103) is present in its active site (see Figure 2a), among which TYR16 and ASP103 directly form H-bond interactions with the carbonyl group of the substrate. Fried et al. probed the electric field using a carbonyl-containing molecule 19-nortestosterone (19-NT, Figure 1d) based on VSE spectroscopy [5]. 19-NT is a product analogue whose C=O group has the same location with the carbonyl group of the substrate molecule (Figure 1c). Their experimental studies [5,6] provide evidence that KSI would use the hydrogen-bonding network to impose enormous electric fields onto the carbonyl group of its bound substrate. By comparing the electric fields exerted onto the C=O group in the active site of wild-type (WT) KSI and a series of mutants with their activation free energies, Fried et al. demonstrated that the electric fields exerted by H-bonds had a significant contribution to the catalytic rate and the activation free energy is linearly correlated with the magnitude of the electric field. 

The site-directed mutagenesis approach is widely used in studies on enzymes for altering their catalytic rate, while the point mutation is often created near the active site which may lead to unexpected structural rearrangement, change of the substrate binding state, and sometimes even introduction of excess water molecules. This is usually imperceptible in macroscopic experimental measurement based on VSE spectroscopy. The case of an experimental study [5,6] on KSI may be more specific, considering the fact that point mutations often involve the removal of specific H-bond interactions formed by the active site of KSI and the substrate. A previous study indicates that these H-bonds are short, strong and may provide additional stabilization energy for the substrate [6]. Therefore, it is essential to carry out corresponding theoretical investigation to confirm the interpretation of the experimental results and explore more detailed worthy information.

Molecular dynamic (MD) simulations are now widely utilized in the study of proteins or other macromolecular systems to reveal more detailed information at the atomic level. Molecular mechanics (MM) force fields based on empirical potentials such as Amber, CHARMM, OPLS, etc. are still the predominant choices in MD simulations due to their inexpensive computational costs. However, despite the great success in applications of the classical force fields, they still have limitations in giving an accurate description of the electrostatic fields of H-bonds in proteins [5,12,16,23,24,25,26,27,28]. More specifically, in the case of KSI, whose active site forms strong and short H-bonds with the substrate, the study [5] by Fried et al. shows that there was a large gap in the electric fields between the calculated value based on a MD simulation using the standard Amber force field and the experimental measurement.

Previous works [12,16,23,24,26,27,28] have demonstrated that QM effects (including electronic polarization and charge transfer) are important for the accurate description of hydrogen bonding interaction in proteins. Therefore, in this study, we employ ab initio quantum mechanics/molecular mechanics (QM/MM) MD simulations to investigate the electric field at the active site of KSI. The key H-bonds are treated by the QM method. Based on the fragment-based QM calculations on electric fields, the correlation between the electric field and the activation free energy is validated from the theoretical perspective, and new physical insights obtained from the accurate simulation of electric fields in proteins are discussed.

## 2. Computational Approaches 

### 2.1. QM/MM MD Simulations of the Wild-Type and Mutants of KSI

The initial model of KSI bound with 19-NT used in this study was taken from the X-ray structure (PDB id: 5KP4) [6]. The mutagenesis tool in the PyMOL [29] program was used to create mutated KSI structures. Three mutated structures (D103N, Y16S, and D103L) were selected in this study. For all protein structures, the hydrogen atoms were added using the LEaP [30] module of the AMBER program. The amine groups of all Lys and Arg residues were protonated, while the carboxylic groups of all Asp and Glu residues were deprotonated, except that Asp103 were left neutral and protonated according to previous studies [5,6]. HIS residues were left neutral and protonated at the N_δ1_ or N_ε2_ position, based on local electrostatic environment. The N-terminal and C-terminal were protonated and deprotonated, respectively. Force field parameters for 19-NT were obtained using the ANTECHAMBER [31] module based on the generalized Amber force field (GAFF) [32] with HF/6-31G* restrained electrostatic potential (RESP) charges [33]. The protein was placed in a periodic rectangular box of TIP3P water molecules. The distance from the surface of the box to the closest atom of the solute was set to 22 Å. Counter ions were added to neutralize the system. Before the QM/MM MD simulation, a series of minimizations using the Amber ff99SB force field were first performed to relax the system. Firstly, the protein atoms were constrained to their initial structure, and only the solvent molecules were optimized. Secondly, the hydrogen atoms of the protein and solvent molecules were relaxed, and the rest of the atoms of the protein were constrained to their initial structures. Thirdly, all of the atoms of the protein and solvent were energy-minimized until convergence was reached. Then the system was brought to room temperature (300 K) at 100 ps with the protein being constrained. The time integration step was 2.0 fs, and the SHAKE [34] algorithm was applied to maintain all hydrogen atoms in reasonable positions. The particle-mesh Ewald [35] method was used to treat long-range electrostatic interactions, and a 10 Å cutoff for the van der Waals interactions was implemented. Langevin dynamics [36] was applied to regulate the temperature with a collision frequency of 1.0 ps^−1^. 

After heating, 40 ps QM/MM MD simulations were performed for WT and for all mutants of KSI. The side chains of residues 16 and 103, and the ring containing the C=O_19-NT_ probe of 19-NT were partitioned into the QM region (see Figure 2) and treated using the M06-2X functional [37] with the 6-31G** basis set, because previous studies have shown that the M06-2X functional could give good description of the hydrogen-bonding interactions [38,39]. The dangling bonds across the QM/MM boundary were treated by the link atom (hydrogen atom) method [40]. The rest of the protein and 19-NT were partitioned into the MM region and described by the Amber ff99SB force field. The TIP3P model was utilized for water molecules. A 25 Å cutoff was used to treat the QM/MM electrostatic interactions. A time integration step of 1.0 fs was adopted, and trajectories were generated with structures being saved every 5 fs. All MD simulations were performed with the AMBER12 program [30]. A previous work [41] by Latouche et al. demonstrated that the Gaussian program is able to perform a QM/MM simulation with good accuracy, and thus the sander module with an interface to the Gaussian09 program [42] was used to carry out the QM/MM MD simulation. 

### 2.2. Calculations of the Electric Field with the Amber ff99SB Force Field

The saved structures from 40 ps QM/MM MD simulation (8000 configurations in total) were used to calculate the electric field along the C=O_19-NT_ bond using the charge model of the Amber ff99SB force field. The electrostatic potentials at atoms C and O of C=O were firstly calculated using the following expression:

(2) $$\varphi \left(\vec{r}\right)=\frac{1}{4\pi \varepsilon_{\mathrm{eff}}}\underset{i}{\sum }\underset{j\epsilon i}{\sum }\frac{q_{ij}}{\left|\vec{r}-{\vec{r}}_{ij}\right|}$$

where *i* runs over all residues of protein, waters (around 8860 water molecules) and 19-NT, where the contribution to electrostatic potentials from the probe C=O_19-NT_ and the ring containing the C=O_19-NT_ group was removed to avoid non-physical values, *j* is the atom number in residue *i*, $q_{ij}$ and ${\vec{r}}_{ij}$ denote the atomic charge and position vector of atom *j* in residue *i*, $\vec{r}$ is the position vector of atoms C or O of C=O_19-NT_, and $\varepsilon_{\mathrm{eff}}$ is the effective dielectric constant, which was set to 1.0 in all calculations, since the explicit water model was utilized [43]. The mean electric field along the C=O_19-NT_ bond is obtained as follows:

(3) $$\vec{F}\left({\vec{r}}_{\mathrm{CO}}\right)=\frac{\varphi \left({\vec{r}}_{C}\right)-\varphi \left({\vec{r}}_{O}\right)}{\left|{\vec{r}}_{\mathrm{CO}}\right|}$$

where $\varphi \left({\vec{r}}_{C}\right)$ and $\varphi \left({\vec{r}}_{O}\right)$ are calculated electrostatic potentials at the positions of the C and O atoms of C=O_19-NT_, respectively, using Equation (2). $\left|{\vec{r}}_{\mathrm{CO}}\right|$ is the C=O_19-NT_ bond length.

### 2.3. Calculations of the Electric Field with the EE-GMFCC Method

The electrostatically embedded generalized molecular fractionation with conjugate caps (EE-GMFCC) method was employed to perform full QM calculations of electric field in KSI. The detailed description of the EE-GMFCC method can be found in our previous works [44,45,46,47,48,49,50,51,52,53,54,55]. The calculations of the electrostatic potential by the EE-GMFCC method are the same as our previous work [16]. Specifically, a protein was decomposed into a number of individual fragments in the unit of an amino acid by cutting through the peptide bond, as illustrated in Figure S1 of the Supplementary Materials. Hydrogen atoms were used to saturate the dangling bonds after cutting. Generalized concaps (Gconcaps) are utilized to capture the short-range QM effect between two non-neighboring residues that are spatially in close contact. QM calculations of all fragments were embedded in the electrostatic field of the point charges representing the remaining atoms of the protein. The point charge model is taken from the Amber 94 force field [56]. The expression of the EE-GMFCC approach for calculating the electrostatic potential of a protein at any point $\vec{r}$ is given by:

(4) $$V\left(\vec{r}\right)=\overset{N-1}{\underset{i=2}{\sum }}V\left(\vec{r}\right)({\mathrm{Cap}}_{i-1}^{*}A_{i}{\mathrm{Cap}}_{i+1})-\overset{N-2}{\underset{i=2}{\sum }}V\left(\vec{r}\right)({\mathrm{Cap}}_{i}^{*}{\mathrm{Cap}}_{i+1})\;+\underset{\begin{array}{c} i,j>i+2\\ \left|R_{i}-R_{j}\right|\leq \lambda \end{array}}{\sum }{\left[V{\left(\vec{r}\right)}_{ij}-V{\left(\vec{r}\right)}_{i}-V{\left(\vec{r}\right)}_{j}\right]}_{\mathrm{QM}}$$

where *N* denotes the number of residues in the protein, *i* and *j* represent the residue number.${\mathrm{Cap}}_{i-1}^{*}A_{i}{\mathrm{Cap}}_{i+1}$ and ${\mathrm{Cap}}_{i}^{*}{\mathrm{Cap}}_{i+1}$ are the capped fragment and conjugate cap respectively. $V{\left(\vec{r}\right)}_{ij}-V{\left(\vec{r}\right)}_{i}-V{\left(\vec{r}\right)}_{j}$ represents the two-body QM contribution to the electrostatic potential from residues *i* and *j* (Gconcap) whose closest distance is less than a predefined threshold λ for including the short-range QM effect. The atomic structures of the capped fragment, the conjugate cap, and Gconcap are illustrated in Figure S1. The distance threshold λ of 4.0 Å was adopted in this study for the calculation of the electrostatic potential, based on our previous benchmark study [16,46,55]. The direct contribution to the electrostatic potential from 19-NT was not included in the QM calculation using Equation (4). Nevertheless, the two-body QM contribution that involves 19-NT was taken into account. The calculated time-average electric field of 19-NT (the contribution to the electrostatic potentials from the probe C=O_19-NT_ and the ring containing the C=O_19-NT_ group was removed) and solvent with the classical charge model is used to amend the QM results. The electric field exerted onto the C=O bond from 19-NT was computed using Equation (3), while the points where the electrostatic potentials were calculated are slightly off the atomic centers along the C=O chemical bond. Considering the expensive cost of the QM method, the single-snapshot approximation was adopted for the EE-GMFCC calculation of electric fields in KSI. Previous studies [16,57] have demonstrated that the single-snapshot approximation implicitly took the conformational sampling of the protein structures into account. In this study, the time-average electric field was initially calculated with the classical charge model from the force field. Subsequently, the structure whose electric field is closest to the time-average value was utilized for the EE-GMFCC calculation. 

### 2.4. Classical MD Simulation and Calculation of the Electric Fields

For comparison, 2 ns classical MD simulations using the Amber ff99SB force field were also performed for WT KSI and three mutants (D103N, Y16S, and D103L), to calculate the electric field exerted onto the C=O group in 19-NT. In the classical MD simulation, the protein was placed in a periodic rectangular box of TIP3P water molecules, while the distance from the surface of the boxer to the closest atom of the solute was set to 12 Å. Counter ions were added to neutralize the system. The minimization and heating steps were the same as the equilibrium simulation for the previous QM/MM MD simulation. After that, a 2 ns MD simulation was performed to generate the trajectories for electric field calculations. The calculation of electric field was using Equations 2 and 3 with the charge model of the Amber ff99SB force field.

## 3. Results and Discussion

### 3.1. The QM Effect is Important for Description of the Electrostatics in H-Bonding Environment

The modeling of the H-bonds in biomolecules with an empirical method is very challenging because it involves important QM effects such as electronic polarization, charge transfer, and the many-body interaction. Many previous studies [12,23,24,26,27,58] have demonstrated that conventional force fields cannot accurately describe the conformations of H-bonds in MD simulations. The strong and short hydrogen bonds between the active site of KSI and its substrate have been observed [6]. In a previous work [5] by Fried et al., classical MD simulations of the KSI·19-NT complex based on Amber force field were carried out to calculate the electric field that exerted onto the C=O probe in 19-NT, and they found that the calculated electric field was much smaller than the value estimated from the experimental VSE spectra based on Equation (1). 

To investigate why the conventional force field could not accurately describe the electric field in the H-bonding environment, we performed both QM/MM and classical MD simulations to calcualte the electric field for wild-type KSI and three mutants. The calculated electric fields based on classical MM simulations (Amber ff99SB) for all proteases are given in Table S1 and Figure S2 of the Supplementary Materials. First, we take WT KSI, and D103N mutant as typical examples (since there are both two H-bonds between residues 16 and 103, and the substrate in their active sites, which would make calculation of the electric field more challenging), and their calculated electric fields based on QM/MM and classical MM simulations are listed in Table 1. From the experimental VSE spectra measured by Fried et al. [5], extremely large electric fields on C=O group of 19-NT were exerted by the wild-type KSI and D103N mutant. The obtained electric fields that the C=O group experiences based on the observed VSE spectra are −144 and –134 MV/cm for WT and D103N, respectively. The calculated time-averaged electric fields from classical MM simulations with the Amber ff99SB charge model are −100 and −66 MV/cm for WT and D103N, respectively (see Table 1), both of which are much smaller than the experimental values. 

Previous works [12,13,57,59] have shown that the calculated electric field is very sensitive to the local chemical environment. The accurate conformational sampling of the key residues plays an important role in the calculation of the electric fields. Considering that both residues 16 and 103 form H-bond interactions with the C=O probe of 19-NT in WT and D103N, there must be significant QM effects in such a hydrogen bonding environment. In QM/MM MD simulations, the QM region includes the two H-bonds formed by the C=O probe, and residues 16 and 103 (see Figure 2), and the generated trajectories were utilized to calculate the electric field with the charge model of the Amber ff99SB force field. The results are marked as “QM/MM-FF” in Table 1. As compared to the results from the MM MD simulation, the calculated electric fields of “QM/MM-FF” for WT and D103N become much closer to the experimental values. 

**Figure 3:**
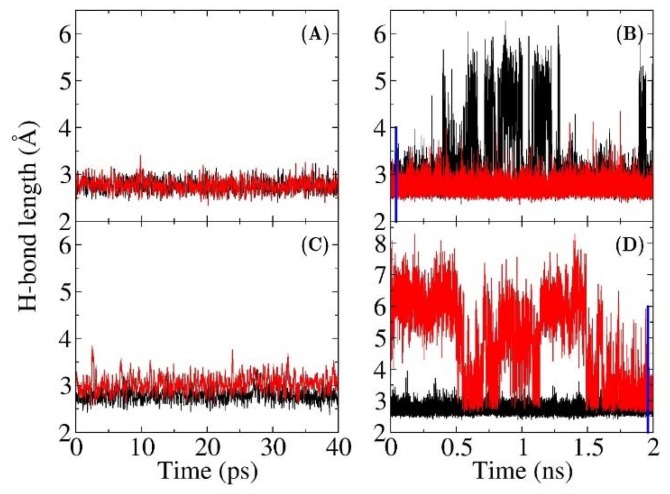
Hydrogen bond length plotted as a function of the MD simulation time. (**A**) and (**B**) are QM/MM and MM simulations, respectively, for the wild type KSI, (black: between OH@Tyr16 and CO@19-NT; red: between OH@Asp103 and CO@19-NT). (**C**) and (**D**) are QM/MM and MM simulations, respectively, for the D103N mutant, (black: between OH@Tyr16 and CO@19-NT; red: between NH@Asn103 and CO@19-NT). The hydrogen bond length is defined by the distance between two O atoms, or the distance between N and O atoms. Blue lines are used to indicate the first 40 ps for WT and the last 40 ps for D103N in (**B**) and (**D**).

The electric fields in both QM/MM-FF and MM models were calculated using the same charge model of Amber ff99SB force field. Hence, the difference in the results is caused by the discrepancy of sampling from QM/MM and MM MD simulations. To reveal the underlying differences at the atomistic level, the distances between two non-hydrogen atoms of the hydrogen bonds between the C=O_19-NT_ probe and residues 16, 103 for WT and D103N are plotted in Figure 3 (the H-bond distances for other two mutants from MM and QM/MM MD simulations are shown in Figures S3 and S4 of the Supplementary Materials, respectively). H-bond lengths are defined as the distances of O_TYR16_⋯O_19-NT_ and O_ASP103_⋯O_19-NT_ in wild-type KSI, and O_TYR16_⋯O_19-NT_ and N_ASN103_⋯O_19-NT_ in the D103N mutant. The hydrogen bond is supposed to be broken when the distance is greater than 3.5 Å. As shown in Figure 3, the H-bond lengths of these two key hydrogen bonds were mainly below 3.5 Å in both the QM/MM MD simulations of WT and D103N. In the MM MD simulation of WT, the O_ASP103_⋯O_19-NT_ distance was mostly shorter than 3.5 Å, but the O_TYR16_⋯O_19-NT_ distance was sometimes very large (greater than 5 Å). Furthermore, in the MM MD simulation of D103N, the O_ASN103_⋯O_19-NT_ distance sometimes also became very large (greater than 5 Å), while the O_TYR16_⋯O_19-NT_ was mainly below 3.5 Å. However, the time scale of the MM simulation was much longer than that of QM/MM simulation. In the 40 ps time duration of the MM MD simulations for WT and D103N, the lengths of two hydrogen bonds could be both shorter than 3.5 Å, e.g., the first 40 ps of MM MD simulation of WT and the last 40 ps of MM MD simulation of D103N (which are indicated with blue lines in Figure 3b,d). For comparison, these 40 ps MD simulations were also selected to calculate the electric fields, and the results were −105 and −71 MV/cm for the WT and the D103N variant, respectively, which were slightly larger than those from the 2 ns MD simulations of −100 (WT) and −66 (D103N) MV/cm. However, the results were still much smaller than those from QM/MM MD simulations of −124 (WT) and –93 (D103N) MV/cm. It resulted from the fact that the hydrogen bonds had different distributions of hydrogen bond lengths in the QM/MM and MM MD simulations. Therefore, the QM interaction was important in stabilizing the hydrogen bonds, which was critical to preserving the local chemical structure of proteins. 

The charge model of the Amber ff99SB force field is derived from the HF/6-31G* calculations by fitting the molecular electrostatic potentials (ESP) of small model peptides, which does not include the electronic polarization and charge transfer effects for a specific chemical environment in folded proteins [33,56]. This mean-field-like charge model is not capable of accurate description of the electrostatics in proteins. Therefore, applying the more sophisticated QM method in simulations of molecular properties is highly needed. To overcome the scaling problem of conventional QM methods, the linear scaling EE-GMFCC method was utilized to calculate the electric fields in the active site of WT KSI and D103N mutant. The EE-GMFCC method has a high computational accuracy, which accounts for almost all of the QM effects (electronic polarization, charge transfer and the many-body effect) in a folded protein. However, it is still computationally demanding to obtain the converged time-average electric field with the EE-GMFCC approach, and thus the single-snapshot approximation was adopted for EE-GMFCC calculations. The QM calculations could provide the insight of the role of QM effect in the calculation of the electric fields by comparison with the force field, and it could also be used in the assessment of the results of the force fields. The single-snapshot approximation was based on the trajectories from QM/MM MD simulations. The time-average electric field (the red line in Figure S5 of the Supplementary Materials) was firstly calculated with the charge model of the force field using the conformations generated by QM/MM MD simulations (the calculated electric field as a function of simulation time is shown in Figure S5). The structure whose electric field is closest to the time-average value (the green cycle in Figure S5) was utilized for subsequent EE-GMFCC calculations. Comparing with the results of QM/MM-FF, the calculated electric fields with the EE-GMFCC method (denoted as “QM/MM-QM” in Table 1) became very close to the experimental data for both WT and D103N. Therefore, the QM effects play significant roles in both the dynamic sampling of the protein structures and the direct calculation of the electric fields.

### 3.2. Calculation of Electric Fields

**Figure 4:**
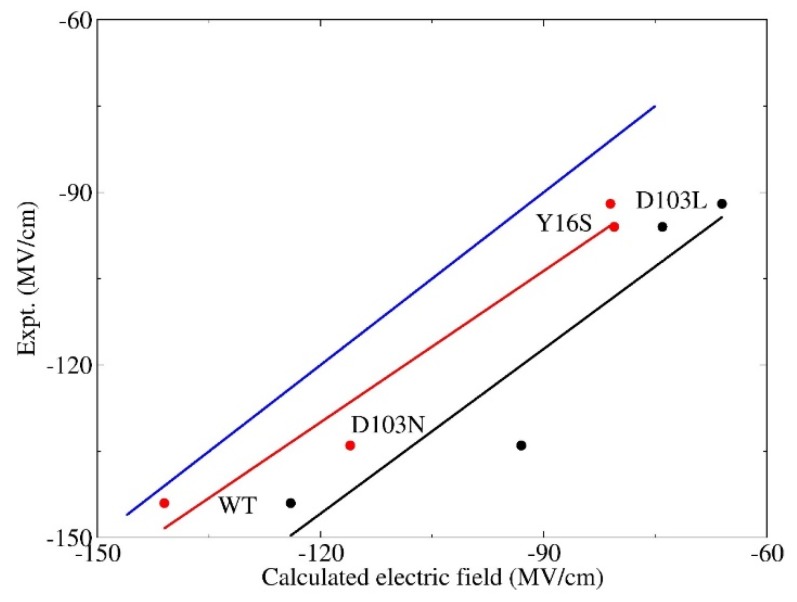
Correlation of the electric fields between the experimental results and theoretical calculations for wild-type and three variants (namely, D103N, Y16S, and D103L). The experimental values were obtained from Fried et al. [5]. The snapshots were taken from QM/MM MD simulations. The results calculated by EE-GMFCC are marked in red. The results calculated by the Amber ff99SB force filed are marked in black. The best-fit lines for these two methods are given in the same colors with *R*^2^ = 0.96 (EE-GMFCC) and 0.91 (Amber ff99SB), respectively. The blue line represents the strict correlation curve.

It is essential to account for the QM effect in simulations of the electric field in the H-bond environment. In this section, the electric fields that the C=O_19-NT_ probe experiences in the wild-type and three mutants (namely, D103N, Y16S, and D103L) are calculated with the EE-GMFCC method and the empirical charge model of Amber ff99SB, respectively, based on the conformations sampled from the QM/MM MD simulations. The experimental work [5] by Fried et al. shows that there are distinct blue-shifts in the observed C=O_19-NT_ VSE spectra, and increases of enzymatic unimolecular free energy barrier of these three mutants with reference to the WT, making them ideal candidates for theoretical investigation of the relationship between the electric field and the activation free energy. The correlation between the calculated electric fields using the EE-GMFCC method with single-snapshot approximation and the experimental values are plotted in Figure 4. For comparison, the calculated electric field using the Amber ff99SB force field is also given in Figure 4. As can be seen from the figure, the predicted electric fields of the WT and three mutants using Amber ff99SB were all much smaller than the experimental results with a systematic shift. However, the calculated electric field versus the experimental measurement showed a good linear relationship with the correlation coefficient *R*^2^ = 0.91. The computed electric fields with the EE-GMFCC method for the WT and the three mutants were consistently closer to the experimental data, as compared to those of Amber ff99SB. It also showed a good linear relationship between the computed electric field and the experimental observations with the correlation coefficient *R*^2^ = 0.96 for EE-GMFCC. Therefore, the trends in computed electric fields for WT and three mutants were both in good agreement with the experiment for EE-GMFCC and Amber ff99SB. It has been discussed in Section 3.1 that the calculated electric field which C=O_19-NT_ experiences becomes larger when the QM effect is taken into account. When the electric field becomes larger, the C=O_19-NT_ group would have stronger interactions with the hydrogen bonding residues, which explains why the QM/MM simulation could better maintain the H-bonds between residues 16, 103, and C=O_19-NT_ than the classical MD simulation. 

The predicted electric fields by EE-GMFCC are still smaller than the experiment for these four systems. There are two possible reasons for this: first, the protocol for the EE-GMFCC calculation did not include explicit conformational sampling at the QM level, due to the expensive computational cost, and only one plausibly representative structure was selected; second, the experimental electric fields estimated from the VSE spectra were based on the linear relationship of Equation (1), which may be overestimated by around 10% owing to neglecting higher-order terms. This has been demonstrated in the previous work based on the ab initio QM calculations of the vibrational frequency of the probe under the external electric field [5]. However, by comparison of the electric fields between the theoretical calculations and experimental measurement, we confirmed that the computed electric fields with the EE-GMFCC method approximately reproduced the experimental observed electric fields in the active site of KSI within an acceptable error range for WT and the three mutants. On the other hand, the Amber ff99SB charge model could also give good relative changes of the electric field despite the discrepancy in the absolute values as compared to the experimental results. 

### 3.3. Electrostatic Contribution to Catalysis in KSI

**Figure 5:**
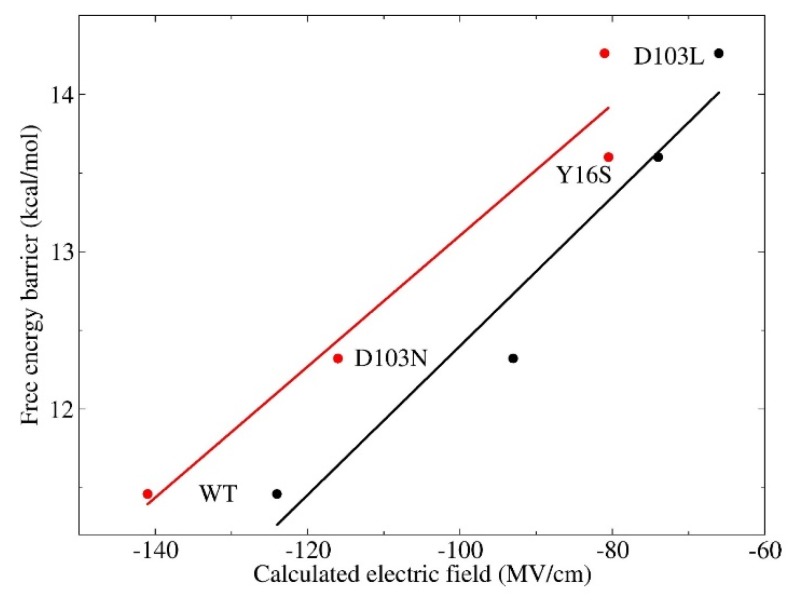
Correlation between the calculated electric fields and the free energy barriers for wild-type and variants of KSI. The experimental values (the activation energies of wild-type KSI and its variants) were obtained from Fried et al. [5], which were estimated from the experimental catalytic rate constant of *k*_cat_ by $\Delta G=-RT\ln\left[k_{\mathrm{cat}}/\left(k_{B}T/h\right)\right]$, where *T* = 293 K, *R* is the ideal gas constant, *k*_B_ is the Boltzmann constant, and *h* is the Planck constant. The best-fit lines are marked in red and black for calculations by EE-GMFCC (*R*^2^ = 0.95) and the Amber ff99SB force field (*R*^2^ = 0.94), respectively.

Accurate modeling the electric field with the QM computational protocol provides the opportunity to critically assess the electrostatic contribution to the catalysis of KSI at the atomistic level. The computed electric fields versus the activation energies of the WT and three mutants (The activation energies of wild-type KSI and its variants were obtained from Fried et al. [5], which were estimated from the experimental catalytic rate constant of *k*_cat_ based on the transition state theory [60,61]) are plotted in Figure 5. For comparison, the results based on Amber ff99SB are also plotted in this figure. Linear correlations between activation energies and the electric fields are observed for both EE-GMFCC and Amber ff99SB with the correlation coefficients (*R*^2^) of 0.95 and 0.94, respectively. It has been suggested that the strong H-bonds between the residues 16 and 103, and the substrate at the active site of KSI have a significant impact on its catalytic rate [5,6]. The linear correlation relationship between the electric fields and the activation energies indicates that KSI mainly utilizes the enormous electric field exerted by these short H-bonds to facilitate chemical reactions. Hence, the physical basis of the electrostatic catalysis could be clearly explained in the isomerization reaction that KSI catalyzes. The C=O group of the substrate would become negatively charged (C-O^−^), and thus possesses a larger dipole moment due to the charge rearrangement in the step of formation of the intermediate state (see Figure 1a,b). The intermediate state would have a stronger interaction with the protein environment under the action of larger electric field along the C=O bond and become well stabilized. As a result, the formation of the intermediate state is accelerated and the reaction rate is enhanced. The activation free energies in wild-type KSI and its mutants are linearly correlated with the magnitudes of the calculated electric fields, which provides a novel and efficient method for designing enzymes with enhanced functions by increasing the electric field in the active site of the enzyme. 

**Figure 6:**
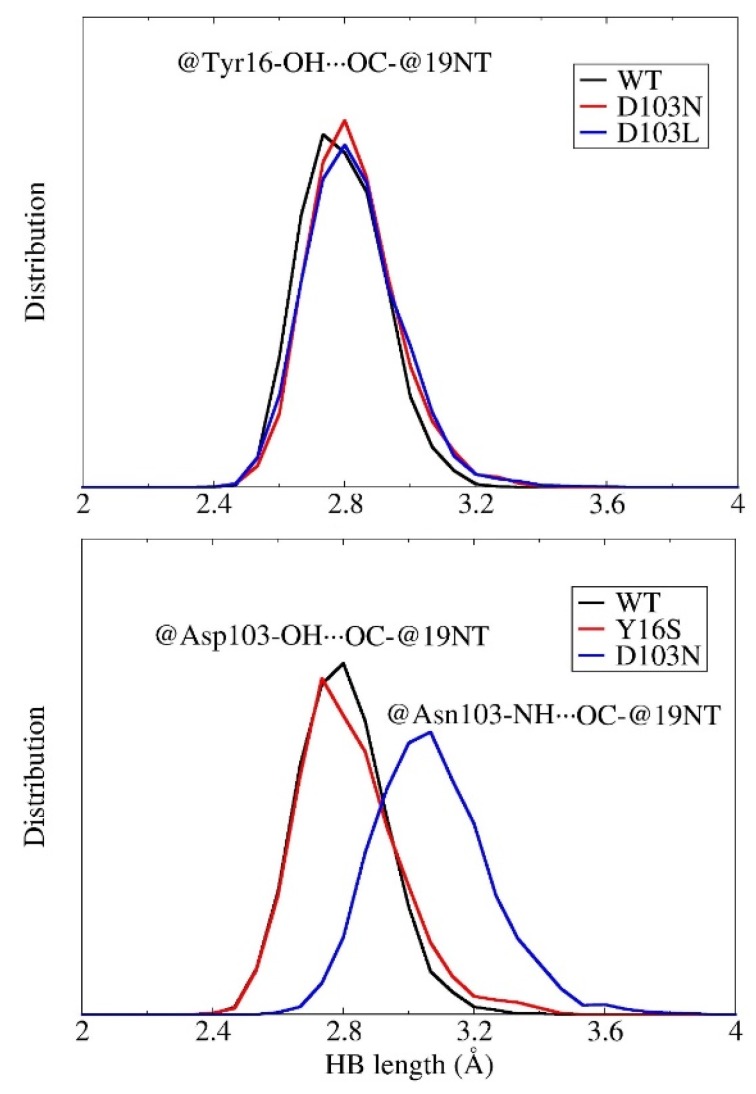
Distribution of hydrogen bond lengths (**A**) between the O atom of Tyr16 (@Tyr16-OH) and the O atom of the C=O group in 19-NT (OC-@19NT), (**B**) between the O atom of Asp103 (@Asp103-OH) and O atom of the C=O group in 19-NT (OC-@19NT) and between the N atom of Asn103 (@ASN103-NH) and O atom of the C=O group in 19-NT (OC-@19NT).

The site-directed mutagenesis approach is widely utilized in theoretical and experimental studies on enzymes. It is worth investigating the structural influences on local chemical environment from the point mutation. In wild-type KSI, the C=O_19-NT_ group forms two strong H-bonds with TYR16 and ASP103; in D103N mutant, one of the H-bonds previously formed between ASP103 and C=O_19-NT_ in WT is replaced by the H-bond formed between ASN103 and C=O_19-NT_, in the Y16S mutant, the H-bond formed by TYR16 and C=O_19-NT_ is removed as compared to WT, and in D103L mutant, the H-bond formed by ASP103 and C=O_19-NT_ is removed. The structures of the H-bond networks for WT and three mutants are shown in Figure S6 of the Supplementary Materials. To investigate whether the change of one of the H-bonds impacts the local hydrogen bonding structure of another, the distributions of H-bond lengths in 40 ps QM/MM MD simulations for WT and three mutants are plotted in Figure 6. As one can see from the figure that the distributions of H-bond length of O_TYR16_⋯O_19-NT_ are very similar in WT, D103N and D103L. Moreover, the distributions of the distance O_ASP103_⋯O_19-NT_ are also very close in the WT and the Y16S mutant. On the other hand, the distribution of H-bond length of O_ASN103_⋯O_19-NT_ in the D103N mutant has an apparent shift to a longer distance as compared to that of O_ASP103_⋯O_19-NT_ in WT, indicating that the H-bond becomes weaker due to mutation of Asp to Asn. These results show that the change of one H-bond has little impact on the H-bonding structure of another in the active site of KSI. 

### 3.4. Decomposition of the Electric Field 

The decomposed time-average electric fields of residues 16, 103, and 40 are listed in Table 2. The calculations of the electric fields utilized the charge model of Amber ff99SB based on the trajectories from QM/MM MD simulations. As discussed in Section 3.2, although the computed electric fields with Amber ff99SB were smaller than the experimental values for WT and the three mutants, the trend in the change of the computed electric field was consistent with the experiment; thus, the calculations based on Amber ff99SB can give reliable qualitative analysis. As shown in Table 2, the electric fields exerted by the residue 16 in WT, D103N, and D103L were approximately equal. Moreover, the electric fields exerted by the residue 103 in WT and Y16S were also very close. It indicates that the change in electric field was mainly caused by the point mutation itself, for these three mutants (D103N, Y16S, and D103L). From the decomposition of electric fields, it also shows that only residues 16, 103, and 40 would exert a large electric field (>20 MV/cm) onto the C=O_19-NT_ group, the electric fields exerted by other residues were all relatively small. The sum of the electric fields from residues 16, 103, and 40 (denoted as “Sum of 3RS” in Table 2) were very close to the total electric field exerted by the protein and solvent. Furthermore, residues 16 and 103 contributed 72–88% of the total electric field for these four systems, implying that these two residues played a leading role in the catalytic rate of KSI. The contributions of the solvent to the total electric field that C=O_19-NT_ experiences were approximately the same in WT and the three mutants, showing that these three mutations had little impact on the solvent environment near the active site. 

## 4. Conclusions

The ab initio QM/MM MD simulations were performed to calculate the electric field mainly exerted by H-bonds in the active site of KSI. By comparing with the simulations using the classical force field, we found that the H-bonds would be dynamically stabilized by the QM electronic polarization effect, which plays an important role in accurate calculations of the electric field. The electric fields of wild-type KSI and three variants were calculated using the EE-GMFCC method and the point charge model of the Amber ff99SB force field based on the configurations from QM/MM MD simulations, respectively. The calculated electric fields by EE-GMFCC were in good agreement with the experimental observations, while the results calculated with the Amber ff99SB force field are all much smaller than the experimental values. In spite of this, based on QM/MM sampling, both the EE-GMFCC method and the Amber ff99SB charge model could provide correct trends in the change of the computed electric fields for the wild type KSI and its three variants. 

By comparing the computed electric fields with the activation energies of these four proteins, a linear correlation relationship between these two physical properties is observed. The detailed structural analysis indicates that the point mutations in the three variants has minor impacts on the overall protein structure and the solvent environment. The change of electric fields in the three variants with respect to the wild type is mainly caused by the mutated residue itself. The electric field that plays a leading role in catalytic rate of KSI is mainly exerted by residues 16 and 103. This study presents a computational protocol for the accurate calculation of the electric field in the active site of the enzyme, and also provides a theoretical foundation supporting the link between electric fields and enzyme catalysis. 

## Figures and Tables

**Table 1:** Calculated electric fields on the C=O group of 19-NT (in MV/cm) with various computational methods. The experimental values were obtained from Fried et al. [5]. “MM” denotes that the calculation is based on the classical force field simulations and calculation of electric fields using the charge model of the Amber ff99SB force field. “QM/MM + FF” denotes that the calculation is based on the QM/MM simulations and the calculation of electric fields with the charge model of the Amber ff99SB force field. “QM/MM + QM” denotes that the calculation is based on the QM/MM simulations and full QM calculation of electric fields using the single-snapshot approach with the EE-GMFCC method.

Model	Exp. [5]	MM	QM/MM + FF	QM/MM + QM
WT	−144	−100	−124	−141
D103N	−134	−66	−93	−116

**Table 2:** The calculated average electric fields on the C=O group of 19-NT (in MV/cm) exerted by residues 16, 103, 40, and the solvent in wild-type and variants (D103N, Y16S, D103L) of KSI. “Sum of 3RS” denotes the sum of the electric field exerted by the three residues 16, 103 and 40. “Sum of ARS” denotes the sum of the electric field exerted by all residues in protein and solvent. “Solvent” denotes the electric field exerted by solvent. The electric field is calculated using the Amber ff99SB charge model from QM/MM MD sampling.

	WT	D103N	Y16S	D103L
RES 16	−47	−46	−2	−45
RES 103	−52	−36	−51	−4
RES 40	−26	−29	−22	−23
Sum of 3RS	−125	−111	−75	−72
Sum of ARS	−124	−93	−74	−66
Solvent	7	10	8	9

## Supplementary Materials

The following are available online. The atomic structures of capped fragment, conjugate cap, and Gconcaps of the EE-GMFCC scheme. The calculated electric field with the charge model of Amber ff99SB as a function of MM and QM/MM MD simulation time. The key H-bond length as a function of MM and QM/MM MD simulation time. Structures of hydrogen bonding network of the active site of KSI for wild type and three mutants D103N, Y16S, and D103L.
