# Supplementary material for: An Ab Initio QM/MM Study of the Electrostatic Contribution to Catalysis in the Active Site of Ketosteroid Isomerase
Source: Molecules. 2018 Sep 20;23(10):2410. doi: 10.3390/molecules23102410 (PMC6222312; doi:10.3390/molecules23102410)
Supplement: Supplementary file 1 [file molecules-23-02410-s001.pdf]

# The Supplementary Materials for

# *Ab Initio* QM/MM Study of the Electrostatic Contribution to Catalysis in the Active Site of Ketosteroid Isomerase

*Xianwei Wang*[1,2] *and Xiao He*[2,3]*

[1] *College of Science, Zhejiang University of Technology, Hangzhou, Zhejiang, 310023, China*

[2] *Shanghai Engineering Research Center of Molecular Therapeutics and New Drug Development, School of Chemistry and Molecular Engineering, East China Normal University, Shanghai, 200062, China*

[3]*NYU-ECNU Center for Computational Chemistry at NYU Shanghai, Shanghai 200062, China*

* To whom correspondence should be addressed: xiaohe@phy.ecnu.edu.cn

**Table S1.** Calculated average electric fields on C=O group of 19-NT (in MV/cm) with the MM method (Amber ff99SB). The experimental values were obtained from Fried *et al.*[1].

| Model | WT | D103N | Y16S | D103L |
|---|---|---|---|---|
| **Exp.[1]** | -144 | -134 | -124 | -141 |
| **ff99SB** | -100 | -66 | -47 | -55 |

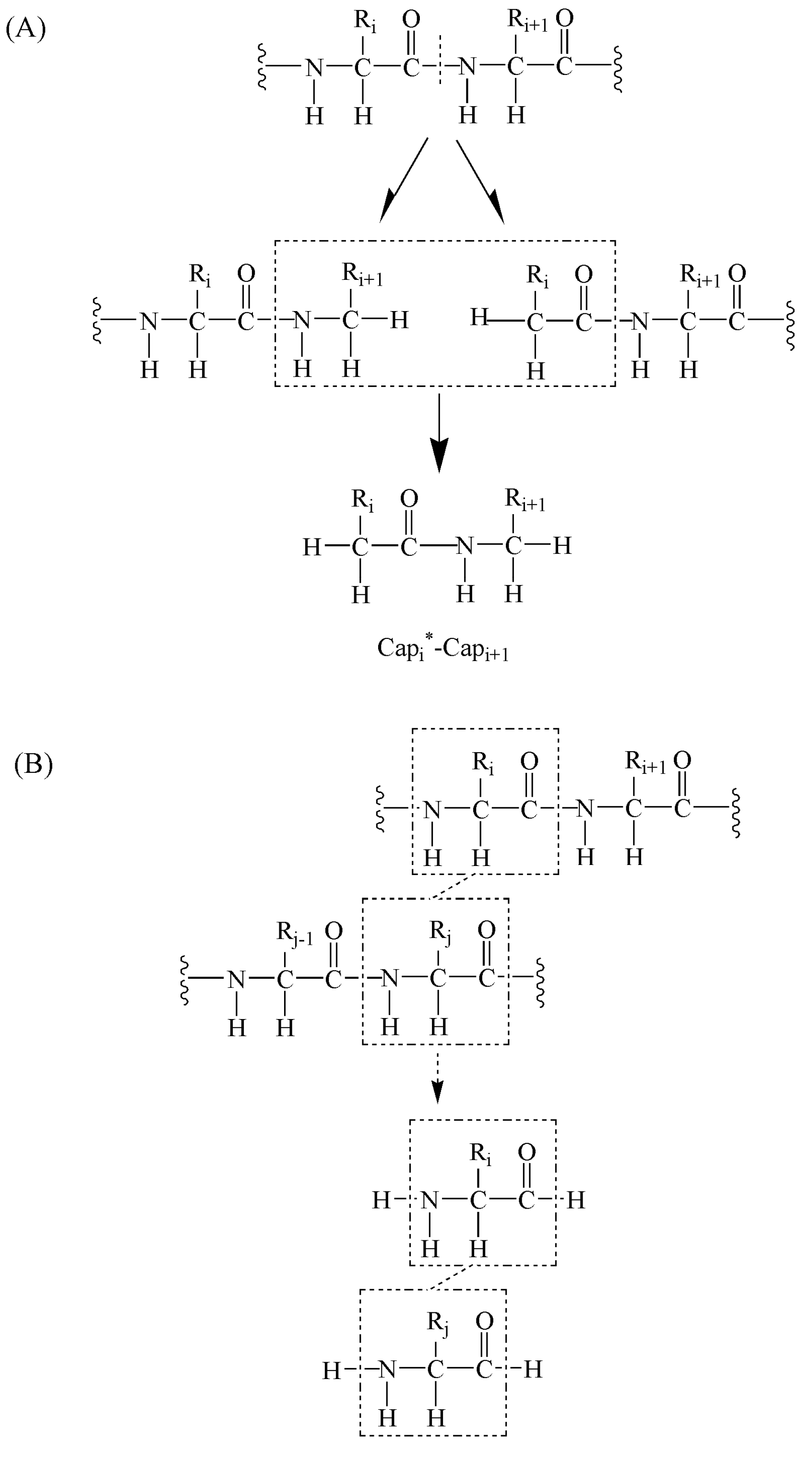


**Figure S1.** (A) The cutting scheme of the EE-GMFCC method. The $\mathrm{Cap}_{i+1}$ and its conjugate $\mathrm{Cap}_i^*$ are used for capping the fragment, where *i* denotes the index of the *i*th amino acid in the given protein. (B) The cutting scheme of the generalized concap (Gconcap) and the atomic structure of the Gconcap.

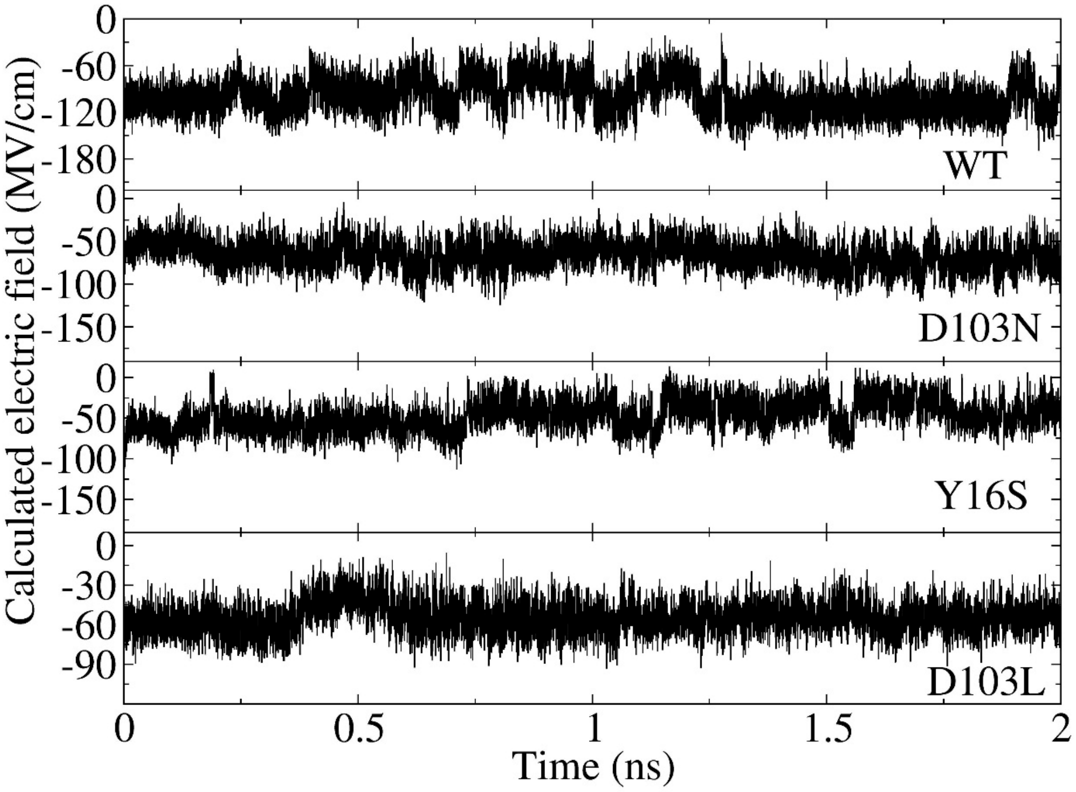


**Figure S2.** The calculated electric field (using the classical charge model of Amber ff99SB force field) as a function of MM MD simulation time for the wild-type KSI and its three mutants (D103N, Y16S and D103L).

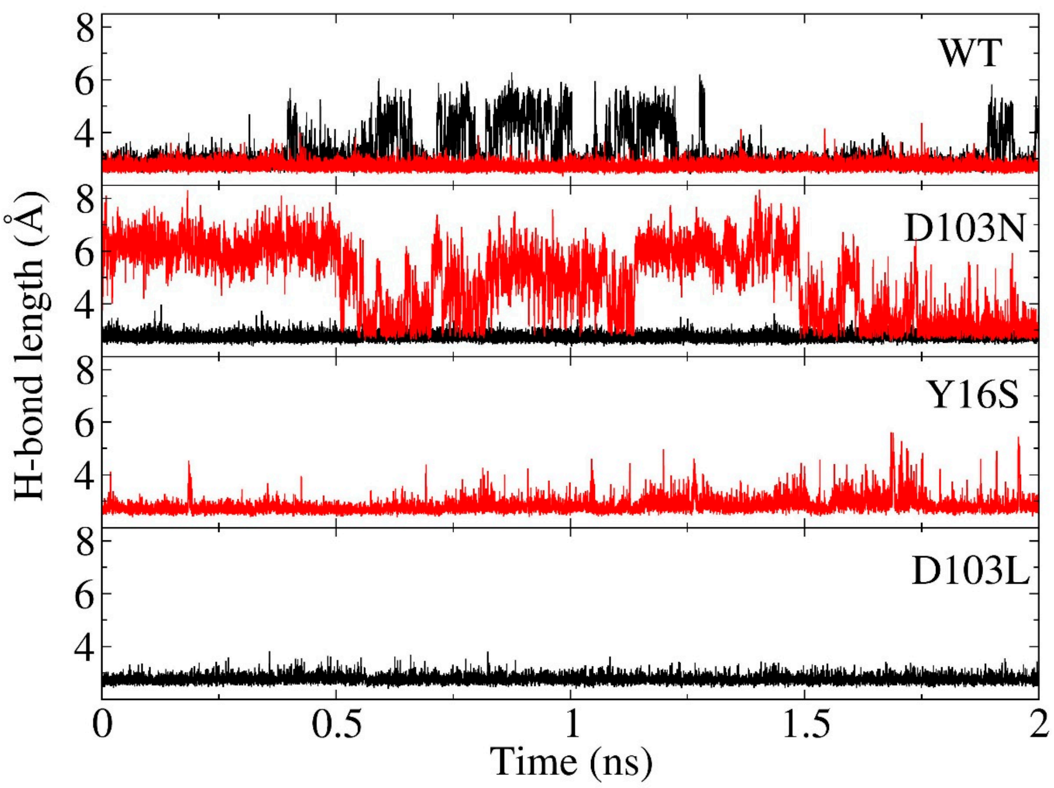


**Figure S3.** The H-bond length as a function of MM MD simulation time for wild-type and three mutants (D103N, Y16S and D103L) of KSI. The black lines represent the H-bond length between residue 16 and 19-NT. The red lines represent the H-bond length between residue 103 and 19-NT.

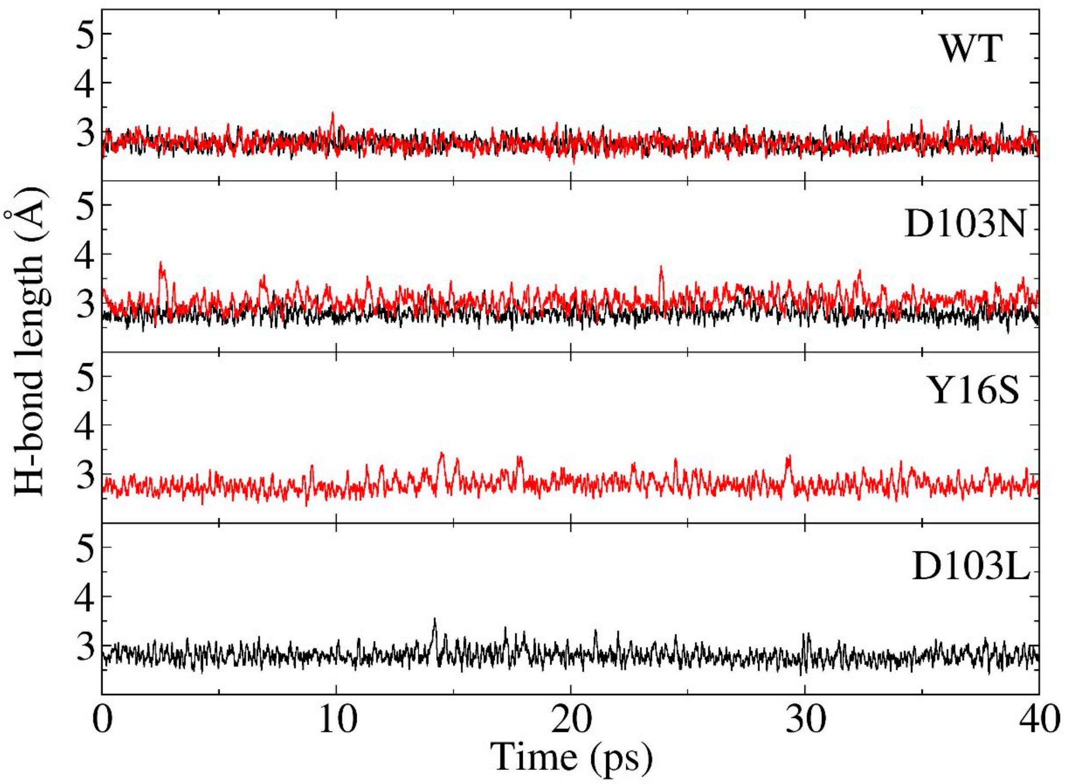


**Figure S4.** The H-bond length as a function of QM/MM MD simulation time for wild-type and three mutants (D103N, Y16S and D103L) of KSI. The black lines represent the H-bond length between residue 16 and 19-NT. The red lines represent the H-bond length between residue 103 and 19-NT.

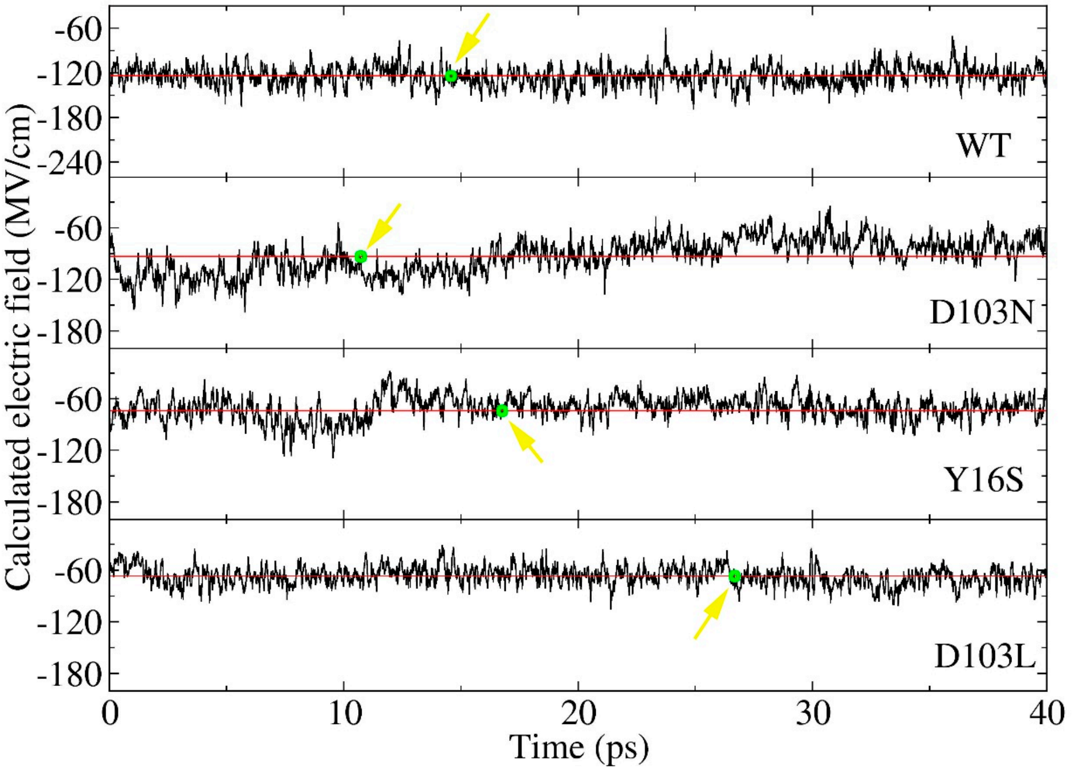


**Figure S5.** The calculated electric field (using the classical charge model of Amber ff99SB force field) as a function of QM/MM MD simulation time for wild-type and three mutants (D103N, Y16S and D103L) of KSI (black line). The red line represents the average value from 40 ps simulation. The green cycle denotes the structure whose electric field is closest to the average value, which is also indicated with a yellow arrow.

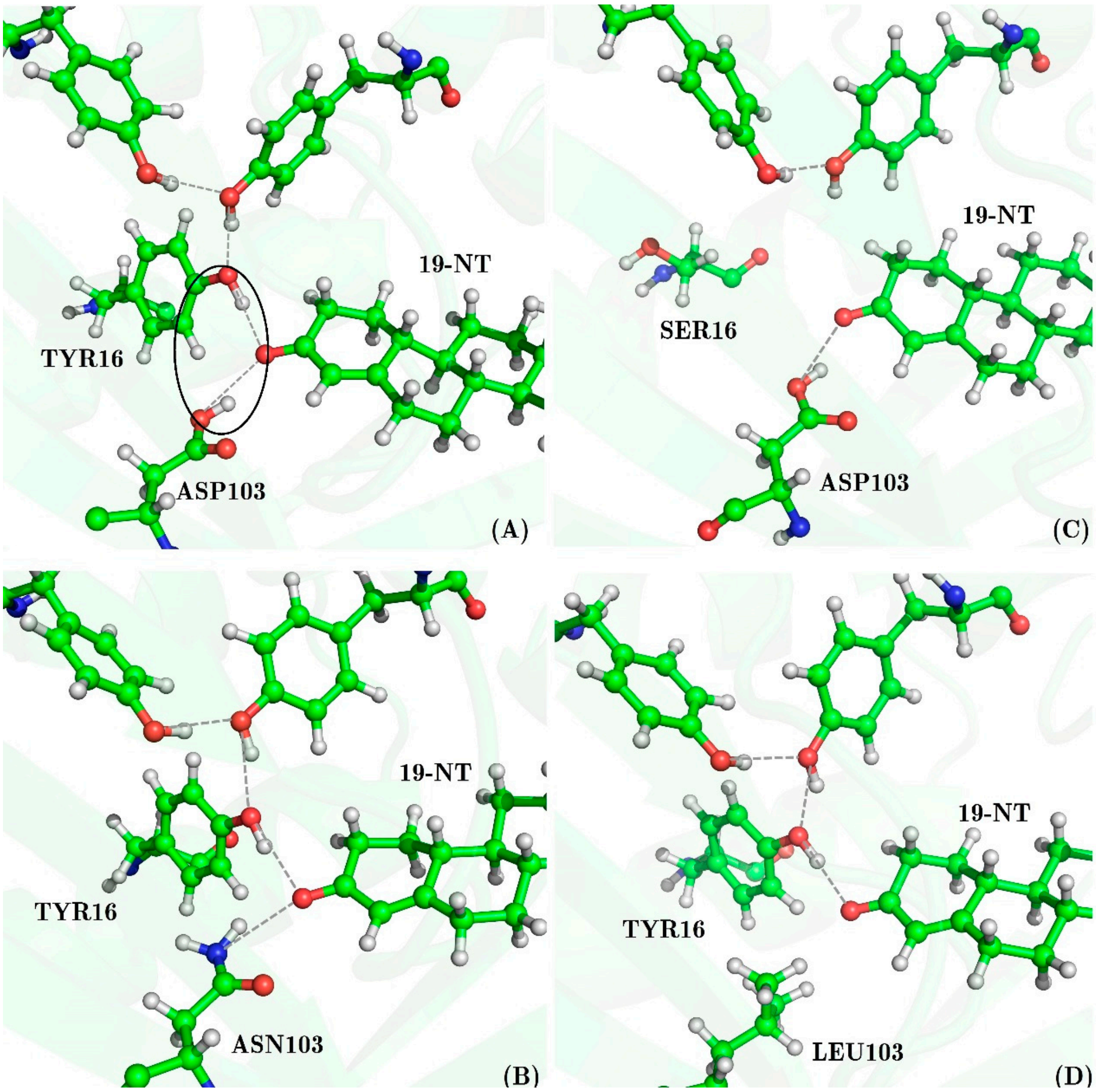


**Figure S6.** Structures of hydrogen bonding network of the active site of KSI for (A) wild type (the *ellipse* highlights the two key H-bonds which play important roles in catalytic strategies of KSI) and three mutants (B) D103N, (C) Y16S and (D) D103L. All structures are selected from snapshots generated by 40 ps QM/MM MD simulations whose H-bond lengths are equal to the most probable value.

## References:

(1) Fried, S. D.; Bagchi, S.; Boxer, S. G. Extreme electric fields power catalysis in the active site of ketosteroid isomerase. *Science* **2014**, *346*, 1510-1514.
